# What can we learn by examining variations in the use of urine culture in the management of acute cystitis? A retrospective cohort study with linked administrative data in British Columbia, Canada, 2005-2011

**DOI:** 10.1371/journal.pone.0213534

**Published:** 2019-03-08

**Authors:** Rachel McKay, Michael Law, Kimberlyn McGrail, Robert Balshaw, Romina Reyes, David M. Patrick

**Affiliations:** 1 School of Population and Public Health, University of British Columbia, Vancouver, Canada; 2 Centre for Health Services and Policy Research, University of British Columbia, Vancouver, Canada; 3 George and Fay Yee Centre for Healthcare Innovation, University of Manitoba, Winnipeg, Canada; 4 LifeLabs, Surrey, Canada; 5 British Columbia Centre for Disease Control, Vancouver, Canada; Rabin Medical Center, Beilinson Hospital, ISRAEL

## Abstract

**Introduction:**

Urinary tract infections (UTI) are common community-based bacterial infections. Empiric antibiotic recommendations are guided by local resistance rates. Previous research suggests that cultures are overused for uncomplicated cystitis, but practice patterns have not been described in detail. Variations in culturing have implications for the interpretation of antibiotic resistance rates.

**Methods:**

We used a retrospective cohort study to analyze variations in urine culturing among physicians, controlling for patient and physician characteristics. We identified all outpatient physician visits among adults and children for cystitis in British Columbia between 2005 and 2011 using administrative data and linked these to laboratory data on urine cultures. Using hierarchical generalized linear mixed models we explored variations in urine culture submissions for cystitis (ICD code 595) and the associations with patient and physician characteristics, stratified by patient sex.

**Results:**

Urine cultures were associated with 16% of visits for cystitis among females and 9% among males, and 59% of visits overall were associated with antibiotic treatment. Older patients, patients with a recent antibiotic prescription, and long term care residents were significantly less likely to have a culture associated with a cystitis visit, whether male or female. Female physicians and physicians with 16–35 years’ experience were more likely to culture, while international medical graduates were less likely–particularly for female visits. Notably, there was substantial unexplained variation among physicians after controlling for physician characteristics: we found a 24-fold variation in the odds of culturing a female UTI between physicians who were otherwise similar.

**Conclusion:**

Individual physicians show substantial variation in their propensity to submit cultures for cystitis visits. Reducing such variation and encouraging appropriate levels of culturing would support effective antibiotic use.

## Introduction

Urinary tract infections (UTI) affect approximately 10% of women over the age of 18 every year, and about 60% of women will experience at least one episode in her lifetime [1,2], making UTIs one of the most common types of bacterial infections among outpatients [3]. While more common in women, UTIs affect men as well. UTIs cause considerable discomfort and impact on quality of life [4–6], cost the healthcare system a significant amount (an estimated $2.3 billion in the US in 2010-equivalent dollars) [2,7], and add to the burden of antibiotic exposure which increases individual risk for antibiotic resistant infections as well as population-level spread of resistant bacteria [8].

The microbiology laboratory is an essential partner in both hospital and community-based antibiotic stewardship initiatives. These labs perform the vital clinical function of working up samples to determine the presence of pathogens, and the susceptibility of the pathogen to pharmaceutical compounds when pathogens are found. Laboratories also analyze and publish aggregate trends in antibiotic resistance, which inform empirical treatment recommendations.

*Escherichia coli* is the predominant pathogen involved in UTI [9]. Complicated UTI generally refers to infection present in an individual with comorbidities, urological abnormalities, men, pregnant and postmenopausal women, while most other cases are considered uncomplicated [3]. Current guidelines recommend treating most uncomplicated UTIs empirically, with urine culture recommended only for patients with complicated UTI, recurrent UTIs, acute pyelonephritis, or those at risk of infection with antibiotic resistant organisms [10]. Local resistance rates should inform treatment practices [3]. Knowledge of local resistance rates derives from urine culture testing results. At best, then, the laboratory-based resistance rate could be considered an overestimate of true community circulation, given that they should be based primarily on complicated UTIs–which carry higher risk of resistance [11]. However, practice patterns are largely unknown. Previous studies have suggested poor adherence to UTI diagnosis and management guidelines [12,13], although the primary focus of these studies has been on decisions regarding antibiotic treatment. Understanding current patterns of urine culturing practice will therefore have the potential to inform our approach to community-level antibiotic stewardship.

From the laboratory perspective, urine cultures comprise a large proportion of the workload; one estimate suggested that 19–32% of all submitted cultures in a clinical laboratory are urine cultures from the outpatient setting [14]. Variations in medical practice have been documented for virtually every condition or procedure studied [15], and it is likely that variations would also be seen among urine culture use [16]. However, research is lacking on the extent and potential drivers of variation in urine culture submission.

A better understanding of the use of culture services for UTI and variations in practice will support meaningful discussion around the appropriate and efficient use of laboratory services in the current era of increasing resistance. The objective of this study was to assess factors associated with urine culture for acute uncomplicated cystitis, and to examine variations in culture use across physicians. To accomplish this, laboratory microbiological analysis data from urine cultures was linked with physician visit and antibiotic prescribing administrative data.

## Methods

We undertook a retrospective cohort study using linked administrative data.

### Patient visits

The universal Medical Services Plan (MSP) in the province of British Columbia, Canada (population 4.5 million in 2011) covers all residents [17]. Our cohort includes all individuals (children and adults] with at least one general practitioner (GP] physician visit for cystitis (ICD 9 code 595] between 2005 and 2011, but we restricted the cohort to patients registered with MSP for at least 275 days of any given year (i.e. patients could be eligible for one year, but not another], to ensure stability of residence and adequate follow up potential [18,19]. Urine culture data were obtained from LifeLabs, a private, community-based laboratory serving the province [20]. When a physician orders an outpatient urine culture, the patient is free to provide that culture in any designated facility. Publicly available financial statements indicate that LifeLabs market share comprised between 60–62% of outpatient urine cultures province-wide during the time frame of this study [21] though with some regional variations. Physician visits were linked to antibiotic prescriptions (ATC code J01) dispensed within 3 days following the visit and to urine cultures dated within 4 days following the visit [22]. We assumed that the physical symptoms of cystitis mean that patients will typically present for cultures within one to two days of their physician visit and will rarely delay filling their prescriptions more than one or two days. We allowed an extra day or two to maximize linkages.

We excluded the small fraction of visits associated with more than one antibiotic dispensed on the same day (0.45% of visit records). As we were only looking at cystitis diagnoses, we could not determine whether these multiple antibiotics were intended for the cystitis, or for another infection.

The University of British Columbia’s Behavioural Research Ethics Board approved this study (certificate H13-00878). Individual consent was not required as the data were analyzed anonymously.

### Measures

#### Outcome

The primary outcome was whether a urine culture was associated with the cystitis visit, counting urine cultures that occurred within 4 days following the UTI visit for the same patient and practitioner.

#### Patient and physician variables

At the patient level, age was included as under 15, 15–24, 25–39, 40–54, 55–69, 70–84, and 85 or older. Recent antibiotic use was defined as a filled prescription for any antibiotic in the 6 months preceding the cystitis visit. Long term care facility residence at the time of the cystitis visit was assessed using the Home and Community Care files [23]. The visit was considered to be follow-up for a potential treatment failure if it occurred within 28 days of a previous visit for a cystitis that had been associated with an antibiotic, similar to previous definitions [24]. We identified patients who had been discharged from hospital in the past 30 days using patient hospitalization records [25]. Patients were considered to have diabetes if at least 2 physician visits within a 2-year period, or one hospitalization, with a diagnosis code of diabetes occurred any time after 2002 and before the cystitis visit. As a proxy for patient attachment with their general practitioner (GP), the proportion of visits in the previous three years that were with the same physician as the cystitis visit was calculated [26], and dichotomized at the median value of 7.3% (meaning that 50% of patients in our cohort had more than 7.3% of visits in the past 3 years with the same physician as that which conducted the UTI visit, and 50% had less than 7.3% of visits with this physician).

At the physician level, variables included sex of the practitioner, as well as variables shown in our previous recent work (submitted and under review) to be associated with antibiotic prescribing decisions in the same time period: medical school location (Canada vs. elsewhere), clinical experience (years of practice as of the date of the cystitis visit), and a measure of UTI service within the physician’s practice (number of UTI visits billed in the past 3 months as a time-varying variable). Physician demographic data are maintained in a dedicated file [27], while the practice data were derived from services billed [17].

We also included a regional identifier variable to control for regional variation in LifeLabs coverage and conducted several sensitivity analyses, described below, to address the potential for confounding in this variable.

### Statistical analysis

Generalized linear mixed models with a logit link were used to estimate the log odds of a urine culture being associated with a cystitis visit (i.e., generalized logistic regression). This method recognizes the natural clustering of patient-visits within physicians.

The intra-class correlation coefficient (ICC) was calculated by the latent variable method [28]. The Median Odds Ratio (MOR) represents the median of the theoretical distribution of odds ratios that could be obtained by comparing two randomly chosen patients, with the same covariate values, from two different physicians [29].

Several sensitivity analyses explored the impact of geographic variation of LifeLabs coverage on estimates of other parameter in the model. First, the model was refit without the regional identifier; second, the analysis was repeated, using only the Vancouver Coastal and Fraser health authority regions where LifeLabs has the greatest market share.

SAS statistical software (version 9.4, SAS Institute Inc,. Cary, NC) was used for the analysis.

## Results

During the study period, there were 1,288,696 visits for cystitis, by 595,714 unique patients, seen by 5,825 unique physicians. Male physicians conducted 71% of all visits; two-thirds of visits were conducted by Canadian medical school graduates; just over a quarter (26.1%) of visits were conducted by physicians with 0–15 years of experience, and almost half were conducted by physicians with 16–30 years of experience. Eighty-four percent of these visits were among female patients. Among females, more visits occurred among younger adults, with 56% of female visits occurring among patients between the ages of 15 and 54. In contrast, only 32% of male visits were in this age group. Fifty-nine percent of male visits were among those aged 55 or older, whereas 38% of female visits were. Overall, 58% of cystitis visits were associated with antibiotic prescription (22% with a prescription for ciprofloxacin, 17% with nitrofurantoin, 12% with trimethoprim/sulfamethoxazole) and.15% of visits were associated with a urine culture (Table 1).

**Table 1:** Distribution of visits and urine cultures.

		Females			Males			Total		
		Total # of visits (N)	% of female visits	% with urine cultures	Total # of visits (N)	% of male visits	% with urine cultures	Total # of visits (N)	% of total visits	% with urine cultures
**Overall**		1,040,636	83.5	15.7	198,594	16.5	9.1	1,239,230	—	14.6
**Calendar year**										
** **	**2005**	131,969	12.7	16.7	25,053	12.6	11.4	157,022	12.7	15.9
** **	**2006**	137,069	13.2	16.3	26,390	13.3	10.7	163,459	13.2	15.4
** **	**2007**	143,861	13.8	13.9	27,855	14.0	7.6	171,716	13.9	12.9
** **	**2008**	148,443	14.3	14.3	28,703	14.5	7.6	177,146	14.3	13.2
** **	**2009**	152,304	14.6	15.4	29,638	14.9	8.2	181,942	14.7	14.2
** **	**2010**	161,365	15.5	16.1	29,972	15.1	9.0	191,607	15.5	15.0
** **	**2011**	165,355	15.9	17.1	30,983	15.6	9.3	196,338	15.8	15.9
**Patient age group**										
** **	**0–14**	62,013	6.0	16.1	19,102	9.6	10.9	81,115	6.6	14.9
** **	**15–24**	141,838	13.6	17.8	9,691	4.9	9.8	151,529	12.2	17.3
** **	**25–39**	226,468	21.8	17.6	22,747	11.5	9.8	249,215	20.1	16.9
** **	**40–54**	223,781	21.5	16.7	33,683	17.0	9.8	257,464	20.8	15.8
** **	**55–69**	167,187	16.1	15.1	43,887	22.1	9.4	211,074	17.0	13.9
** **	**70–84**	148,046	14.2	13.0	51,256	25.8	8.1	199,302	16.1	11.7
** **	**85+**	71,303	6.9	9.1	18,228	9.2	6.6	89,531	7.2	8.6
**Potential treatment failure follow-up#**										
** **	**No**	991,303	95.3	15.6	192,846	97.1	9.0	1,184,179	95.6	14.5
** **	**Yes**	49,303	4.7	17.4	5,748	2.9	11.4	55,051	4.4	16.7
**The patient is a long term care resident at the time of the visit**										
** **	**No**	1,005,774	96.7	16.0	189,971	95.7	9.2	1,195,745	96.5	14.9
** **	**Yes**	34,862	3.4	7.5	8,623	4.3	5.2	43,485	3.5	7.0
**Patient has had an antibiotic prescription in the past 6 months**										
** **	**No**	811,225	78.0	16.0	146,851	74.0	9.3	958,076	77.3	15.0
** **	**Yes**	229,441	22.1	14.5	51,743	26.1	8.4	281,154	22.7	13.4
**Diabetes**										
** **	**No**	941,523	90.5	16.1	163,634	82.4	9.4	1,105,157	89.2	15.1
** **	**Yes**	99,113	9.5	12.2	34,960	17.6	7.7	134,073	10.8	11.0
**In hospital within 30 days of prior UTI visit**										
** **	**No**	1,034,191	99.4	15.8	194,163	97.8	9.1	1,228,354	99.1	14.7
** **	**Yes**	6,445	0.6	7.1	4,431	2.2	5.9	10,876	0.9	6.6
**Patient-physician familiarity**										
** **	**Patient has no visits in past 3 years**	34,941	3.4	17.2	7,233	3.6	11.8	42,174	3.4	16.2
** **	**Less familiar (< = 7.3% (median) of patient's visits in past 3 years were with the UTI physician)**	521,199	50.1	16.2	76,037	38.3	8.9	597,236	48.2	15.3
** **	**More familiar (>7.3% of patient's visits in past 3 years were with UTI physician)**	484,496	46.6	15.1	115,324	58.1	9.0	599,820	48.4	13.9
**Physician sex**										
** **	**Female**	317,146	30.5	18.7	41,101	20.7	9.8	358,247	28.9	17.7
** **	**Male**	722,265	69.5	14.3	157,382	79.3	8.8	879,647	71.1	13.4
** **	**missing**	1,225			111			1,336		
**Physician's UTI volume (number of billed visits) in past 3 months**										
** **	**Q1, under 13**	275,222	26.5	13.7	41,593	20.9	8.1	316,815	25.6	13.0
** **	**Q2, 13–24**	272,714	26.2	15.2	47,552	23.9	9.7	320,266	25.8	14.4
** **	**Q3, 25–45**	269,156	25.9	16.5	54,581	27.5	9.4	323,737	26.1	15.3
** **	**Q4, 45+**	223,544	21.5	17.9	54,868	27.63	8.9	278,412	22.5	16.1
**Physician years of clinical experience**										
** **	**0-5yrs**	56,739	5.5	14.6	7,917	4.0	8.1	64,656	5.2	13.9
** **	**6-10yrs**	99,105	9.5	13.7	16,225	8.2	8.1	115,330	9.3	12.9
** **	**11-15yrs**	126,017	12.1	14.9	20,422	10.3	8.7	146,439	11.8	14.0
** **	**16-20yrs**	169,951	16.3	17.6	32,155	16.2	10.1	202,106	16.3	16.4
** **	**21-25yrs**	177,547	17.1	16.9	35,348	17.8	9.2	212,895	17.2	15.6
** **	**26-30yrs**		13.9	15.0	29,166	14.7	144,654	173,820	14.0	13.9
** **	**31-35yrs**	134,938	13.0	16.5	27,536	13.9	10.3	162,474	13.1	15.5
** **	**36-40yrs**	85,072	8.2	15.7	19,536	9.7	9.0	104,355	8.4	14.5
** **	**40+yrs**	46,613	4.5	11.6	10,542	5.3	6.5	57,155	4.6	10.7
**Physician place of medical school**										
** **	**Canada**	685,374	67.2	16.2	132,770	67.9	9.1	818,144	67.4	15.0
** **	**International**	333,878	32.8	14.6	62,770	32.1	8.8	396,648	32.7	13.7
** **	**missing**	21,384			3,054			24,438		
**Antibiotic therapy**										
** **	**Ciprofloxacin**	239,810	23.0	19.4	34,790	17.5	17.8	274,600	22.2	19.2
** **	**Nitrofurantoin**	201,633	19.4	21.2	9,416	4.7	14.1	211,049	17.0	20.9
** **	**TMP-SMX**	136,902	13.2	16.2	13,641	6.9	15.4	150,543	12.2	16.1
** **	**Amoxicillin**	28,474	2.7	16.8	3,424	1.7	11.3	31,898	2.6	16.3
** **	**Cefalexin**	16,674	1.6	17.2	2,361	1.2	11.8	19,035	1.5	16.5
** **	**Norfloxacin**	13,391	1.3	15.3	1,116	0.6	14.6	14,507	1.2	15.2
** **	**Other**	21,409	2.1	13.4	5,971	3.0	9.2	27,380	2.2	12.4
	**No Rx**	382,343	36.7	10.3	127,875	64.4	5.5	510,218	41.2	9.1

*Percent of all visits

^#^Defined as a cystitis visit within 28 days of a prescription for cystitis

### Model results

Table 2 shows the results of the final models. Among female visits, there was a lower probability of culturing with increasing age: children under 15 years had higher odds of culture compared to adolescents 15–24 years old (adjusted odds ratio (aOR) 1.18, 95% CI 1.14–1.22), while adults 85 years and older had lower odds compared to adolescents (aOR 0.69, 95% CI 0.66–0.71). The same pattern was generally apparent among male patients as well. After accounting for all other variables, visits that were likely the result of prior treatment failure were associated with a decreased probability of culturing among female and male patients. Both male and female long-term care residents were less likely to have a culture submitted to LifeLabs than community-dwelling individuals. Recent hospitalization was associated with lower odds of culture among females (aOR 0.73, 95% CI 0.65–0.81), but not among males (aOR 0.96, 95% CI 0.83–1.12). Both females and males with an antibiotic prescription in the past 6 months were less likely to have a culture. We explored whether this last finding was related to prior use of culture subsequently guiding the present treatment plan, but found no strong evidence to support that theory.

**Table 2:** Generalized linear mixed models for the odds of urine culture per cystitis visit. Model 1 includes only patient-level covariates, and model 2 includes both patient- and physician-level covariates. Complete case analysis.

		Model 1				Model 2			
		Female patients		Male patients		Female patients		Male patients	
		OR	95% CI	OR	95% CI	OR	95% CI	OR	95% CI
**Year of visit (reference: 2005)**	**2006**	0.96	0.94–0.98	0.92	0.87–0.99	0.94	0.92–0.97	0.92	0.86–0.99
	**2007**	0.72	0.71–0.74	0.56	0.52–0.60	0.69	0.67–0.71	0.55	0.51–0.59
	**2008**	0.77	0.75–0.79	0.59	0.55–0.63	0.74	0.72–0.76	0.59	0.54–0.63
	**2009**	0.84	0.82–0.86	0.62	0.58–0.66	0.81	0.78–0.84	0.62	0.57–0.67
	**2010**	0.92	0.89–0.94	0.68	0.64–0.73	0.87	0.84–0.90	0.69	0.64–0.74
	**2011**	1.04	1.02–1.07	0.71	0.67–0.76	1.00	0.97–1.04	0.73	0.67–0.79
**Patient age group (reference: 15–24)**	**0–14**	0.98	0.95–1.01	1.18	1.07–1.29	1.18	1.14–1.22	1.32	1.20–1.46
	**25–39**	0.97	0.95–0.99	1.02	0.93–1.12	0.99	0.97–1.01	0.91	0.83–1.00
	**40–54**	0.94	0.92–0.96	1.07	0.98–1.17	0.95	0.93–0.97	0.91	0.83–0.99
** **	**55–69**	0.92	0.89–0.94	1.15	1.05–1.25	0.93	0.91–0.96	0.95	0.87–1.04
** **	**70–84**	0.84	0.82–0.86	1.12	1.02–1.22	0.87	0.84–0.89	0.94	0.85–1.03
** **	**85+**	0.64	0.62–0.66	1.01	0.91–1.12	0.69	0.66–0.71	0.85	0.76–0.95
**Follow up visit**#		1.08	1.05–1.11	1.12	1.02–1.24	0.86	0.84–0.89	0.67	0.61–0.74
**Long term care resident**		0.45	0.43–0.48	0.41	0.36–0.46	0.49	0.46–0.51	0.45	0.40–0.51
**Patient had recent antibiotic prescription†**		0.85	0.84–0.87	0.77	0.74–0.80	0.90	0.89–0.91	0.82	0.79–0.86
**Patient was discharged from hospital in the previous 30 days**		0.66	0.59–0.74	0.91	0.79–1.06	0.73	0.65–0.81	0.96	0.83–1.12
**Diabetes**		0.91	0.89–0.93	0.92	0.87–0.97	0.91	0.89–0.93	0.92	0.87–0.97
**Proportion of patient's visits in last 3 years that were with this physician (reference: >7.3%)**	**Patient had no medical visits in the past 3 years**					1.01	0.97–1.05	1.09	0.99–1.20
	**< = 7.3%**					1.00	0.98–1.02	1.08	1.03–1.13
**Female Physician**						1.62	1.32–1.98	1.37	1.15–1.64
**International medical school graduation location (reference: Canada)**						0.55	0.44–0.69	0.74	0.62–0.89
**Number of years of clinical experience (reference 0–5 years)**	**6-10yrs**					1.07	1.02–1.13	1.12	0.96–1.30
	**11-15yrs**					1.16	1.07–1.25	1.10	0.92–1.32
	**16-20yrs**					1.31	1.20–1.42	1.19	0.98–1.44
	**21-25yrs**					1.31	1.18–1.44	1.20	0.98–1.47
	**26-30yrs**					1.30	1.16–1.46	1.18	0.95–1.46
	**31-35yrs**					1.28	1.12–1.46	1.31	1.04–1.65
	**36-40yrs**					1.17	1.01–1.36	1.14	0.89–1.46
	**40+yrs**					1.20	1.01–1.44	0.91	0.68–1.22
**Physician's UTI volume—number of UTIs seen in the previous 3 months (reference: Q1, <13)**	**Q2, 13–24**					1.07	1.05–1.09	1.15	1.08–1.23
	**Q3, 25–45**					1.16	1.13–1.19	1.14	1.06–1.24
	**Q4, 45+**					1.15	1.11–1.19	1.19	1.09–1.31
**Antibiotic prescribed (reference: No antibiotic prescription)**	**Ciprofloxacin**					2.37	2.33–2.42	4.35	4.15–4.56
	**Nitrofurantoin**					2.22	2.17–2.26	3.11	2.87–3.36
	**TMPSMX**					2.13	2.08–2.18	3.62	3.38–3.87
	**Amoxicillin**					1.73	1.66–1.80	1.87	1.64–2.12
	**Cefalexin**					1.87	1.77–1.97	2.11	1.81–2.46
	**Norfloxacin**					2.17	2.03–2.31	3.09	2.49–3.84
	**Other**					1.64	1.56–1.72	1.86	1.68–2.07
**Number of observations**		1,040,636		198,594		1,015,911		195,050	
**Number of patients**		468,146		108,969		460,419		107,038	
**Number of general practitioners**		5,752		4,865		5,637		4,763	
**Random intercept variance**		11.06		6.23		11.01		4.32	
**Random effect standard error**		0.39		0.29		0.39		0.20	
**Median Odds Ratio**		23.86		10.81		23.68		7.26	
**Intraclass correlation coefficient**		0.77		0.65		0.77		0.57	

^#^ Defined as a cystitis visit within 28 days of a previous cystitis treated with antibiotics

† Defined as any antibiotic prescription within 6 months prior to the cystits visit

Female physicians were significantly more likely to submit a culture for both female and male patients (aOR 1.62, 95 CI 1.32–1.98 and aOR 1.37, 95% CI 1.15–1.64 respectively). International medical graduates were less likely than Canadian graduates to submit a culture. Compared to early-career physicians, those with 16–35 years of experience were generally more likely to culture their patients, although the effect was stronger with female patients. Physicians who saw more UTIs in their practice in the 3 months prior to the visit, generally cultured more.

#### Variation across physicians

The intraclass correlation coefficient (ICC) for the female patient model was 0.77, implying that 77% of the total variation in culturing could be accounted for by differences between physicians. Among male patients, the ICC was slightly smaller, but still large, at 57%. The median odds ratio (MOR) was 23.7 among female visits, and 7.3 among male visits.

#### Sensitivity analyses

Parameter estimates from the model that did not control for Health Service Delivery Area (HSDA) (Table 3) were substantively similar to our original analysis. When we restricted the analysis to the two regions with greatest LifeLabs coverage, there were slightly some differences in the parameter estimates compared to the original analysis, although the directions of effects all remained the same. The small differences in magnitude would not lead to different conclusions in general. The estimated variances of the random effects behaved as expected with larger variances with the exclusion of the HSDA control variable, and smaller variance with restriction to a smaller population of physicians.

**Table 3:** LifeLabs coverage sensitivity analyses. Generalized linear mixed models for the odds of urine culture per cystitis visit. Full model results presented for model that did not control for region, and that was restricted to the broad regions with highest LifeLabs coverage.

		Without controlling for HSDA^1^				Restricted to VCHA^2^ and FHA^3^			
		Female patients		Male patients		Female patients		Male patients	
		OR	95% CI	OR	95% CI	OR	95% CI	OR	95% CI
**Year of visit** **(reference: 2005)**	**2006**	0.94	0.92–0.97	0.92	0.86–0.99	0.94	0.91–0.97	0.91	0.85–0.99
	**2007**	0.69	0.67–0.71	0.55	0.51–0.59	0.61	0.59–0.63	0.49	0.45–0.53
	**2008**	0.74	0.72–0.76	0.58	0.54–0.63	0.63	0.61–0.66	0.51	0.46–0.55
** **	**2009**	0.81	0.78–0.83	0.62	0.57–0.67	0.68	0.65–0.70	0.52	0.48–0.57
** **	**2010**	0.87	0.84–0.90	0.68	0.63–0.74	0.75	0.72–0.77	0.59	0.54–0.64
** **	**2011**	1.00	0.97–1.04	0.72	0.66–0.78	0.84	0.81–0.88	0.61	0.56–0.66
**Patient age group (reference: 15–24)**	**0–14**	1.18	1.15–1.22	1.36	1.23–1.50	1.19	1.14–1.23	1.38	1.24–1.54
	**25–39**	0.99	0.97–1.01	0.93	0.85–1.03	0.99	0.96–1.01	0.94	0.84–1.04
	**40–54**	0.95	0.93–0.97	0.93	0.85–1.02	0.94	0.92–0.97	0.95	0.86–1.05
	**55–69**	0.93	0.91–0.96	0.98	0.89–1.07	0.92	0.89–0.95	0.99	0.89–1.09
	**70–84**	0.87	0.85–0.89	0.96	0.88–1.05	0.84	0.82–0.87	0.96	0.87–1.07
	**85+**	0.69	0.66–0.71	0.87	0.78–0.97	0.67	0.64–0.70	0.88	0.77–0.99
**Follow up visit**^**4**^		0.86	0.84–0.89	0.67	0.61–0.74	0.83	0.80–0.86	0.66	0.59–0.74
**Long term care resident**		0.49	0.46–0.51	0.45	0.40–0.51	0.57	0.54–0.61	0.54	0.48–0.62
**Patient had recent antibiotic prescription**^**5**^		0.90	0.89–0.91	0.82	0.78–0.85	0.90	0.88–0.91	0.80	0.76–0.84
**Patient was discharged from hospital in the previous 30 days**		0.73	0.65–0.81	0.97	0.83–1.13	0.70	0.62–0.80	0.97	0.81–1.15
**Diabetes**		0.91	0.89–0.94	0.92	0.87–0.97	0.92	0.89–0.94	0.92	0.86–0.98
**Proportion of patient's visits in last 3 years that were with this physician (reference: >7.3%)**	**no visits**	1.01	0.97–1.05	1.10	0.99–1.21	1.03	0.99–1.08	1.08	0.97–1.20
	**< = 7.3%**	1.00	0.98–1.02	1.08	1.03–1.14	1.05	1.03–1.07	1.09	1.03–1.15
**Female Physician (reference: male)**		1.62	1.32–1.98	1.53	1.26–1.87	1.55	1.30–1.85	1.49	1.23–1.80
**International medical school graduation location (reference: Canada)**		0.56	0.44–0.69	0.65	0.53–0.79	0.73	0.60–0.88	0.75	0.62–0.92
**Number of years of clinical experience/**	**6-10yrs**	1.07	1.05–1.13	1.12	0.96–1.31	1.00	0.94–1.06	1.09	0.92–1.29
	**11-15yrs**	1.16	1.08–1.25	1.12	0.93–1.35	1.05	0.97–1.14	1.10	0.90–1.34
	**16-20yrs**	1.30	1.19–1.42	1.24	1.01–1.51	1.16	1.06–1.28	1.13	0.91–1.39
	**21-25yrs**	1.31	1.18–1.44	1.27	1.02–1.56	1.19	1.07–1.33	1.19	0.96–1.49
	**26-30yrs**	1.30	1.16–1.44	1.29	1.03–1.62	1.15	1.02–1.31	1.16	0.92–1.47
	**31-35yrs**	1.28	1.12–1.46	1.48	1.16–1.88	1.16	1.00–1.33	1.28	1.00–1.65
	**36-40yrs**	1.17	1.01–1.36	1.30	1.00–1.71	1.03	0.88–1.21	1.10	0.84–1.45
	**40+yrs**	1.20	1.01–1.43	1.09	0.79–1.49	1.12	0.93–1.35	0.98	0.71–1.35
**Physician's UTI volume—number of UTIs seen in the previous 3 months (reference: Q1, <13)**	**Q2, 13–24**	1.07	1.05–1.09	1.17	1.10–1.25	1.06	1.03–1.09	1.12	1.03–1.21
	**Q3, 25–45**	1.16	1.13–1.19	1.17	1.09–1.27	1.14	1.11–1.18	1.12	1.02–1.22
	**Q4, 45+**	1.16	1.12–1.20	1.23	1.12–1.35	1.11	1.06–1.15	1.15	1.03–1.28
**Antibiotic prescribed (reference: No antibiotic prescription)**	**Amoxicillin**	1.73	1.67–1.81	1.86	1.63–2.11	1.68	1.60–1.76	1.74	1.50–2.02
	**Cefalexin**	1.87	1.78–1.97	2.1	1.80–2.45	1.84	1.74–1.95	2.12	1.79–2.52
	**Ciprofloxacin**	2.37	2.33–2.42	4.33	4.12–4.54	2.39	2.34–2.44	4.50	4.26–4.75
	**Nitrofurantoin**	2.22	2.17–2.26	3.08	2.85–3.33	2.19	2.14–2.24	3.12	2.84–3.42
	**Norfloxacin**	2.17	2.03–2.31	3.11	2.50–3.86	2.04	1.87–2.24	3.21	2.41–4.26
	**Other**	1.64	1.56–1.73	1.88	1.69–2.09	1.62	1.53–1.72	1.88	1.66–2.12
	**TMPSMX**	2.13	2.09–2.18	3.59	3.36–3.84	2.13	2.08–2.19	3.70	3.43–3.99
**N observations**	** **	1,018,207		195,429		627,914		128,597	
**Random intercept variance**		11		6.1		6.02		3.56	
**Random effect standard error**		0.39		0.29		0.23		0.19	

^1^ HSDA: Health Service Delivery Area

^2^ VCHA: Vancouver Coastal Health Authority

^3^ FHA: Fraser Health Authority

^4^ Defined as cystitis visit within 28 days of a previous cystitis treated with antibiotics

^5^ Defined as any antibiotic prescription within 6 months prior to the cystitis visit

## Discussion

Rising antibiotic resistance among uropathogens complicates the treatment of urinary tract infections. Culturing a UTI specimen can facilitate treatment through identification of the organism and susceptibility profile; however, given the stability of organism distribution and relative predictability of susceptibility probabilities, there is good evidence that there is no clinical advantage from routinely sending urine cultures from suspected uncomplicated cystitis for testing [30]. Microbiology services support optimal clinical care when used appropriately, and are an essential component of antimicrobial stewardship [31]. Overuse of culturing in uncomplicated UTI may represent potential system waste. However, in complicated cases, where the distribution of organisms and resistance patterns is less predictable, a urine culture can be important–both in terms of targeting the clinical management of the patient with the goal of reducing symptoms quickly, and for minimizing undue selection pressure for antibiotic resistance. Therefore, encouraging the appropriate balance of use is both a patient care and health system issue. This study found large variations between physicians in their propensity to culture, and these variations were larger than the marginal effect of any of our observed characteristics of patients or physicians.

There are three aspects of these findings worth focusing on here. First, there were several patient-level variables that would be expected to be positively associated with urine culturing, that were in fact negatively associated. Particularly among female patients, this analysis found that recent antibiotic use, a history of diabetes, recent hospitalization, and older patients had lower odds of a urine culture, despite these being indications for culture [10,32,33]. This suggests potential underuse of the microbiology resource among those who might benefit, or alternatively, potential overuse among those who likely would not. However, we cannot rule out the possibility that physicians had access to a recent previous culture (for instance, in hospital, or through a different laboratory) that was guiding treatment decisions, or that there was some form of systematic bias in these cases being more likely to be cultured at a laboratory other than LifeLabs. Long term care residents, who carry higher prevalence of resistance in urine isolates, had lower odds of having a culture submitted to LifeLabs; however, this could be because their cases are more likely to be referred to hospital. Further assessment will be needed to explore this.

Second, a number of variables at the physician level were associated with the probability of a urine culture, highlighting the complexity of clinical decision-making. In principle, a patient’s clinical characteristics and medical need should be the only relevant factors in diagnostic and treatment decisions. However, a number of studies have demonstrated variation in physician practice on the basis of the physician’s sex [34], or years in medical practice and international medical graduation [35]. The results of this study support the conclusion that there can be differences in practice associated with these characteristics.

Third, and perhaps most importantly, we have described substantial variations among physicians in the decision to culture for cystitis. A female patient presenting with cystitis to a physician with a higher propensity to culture has a median nearly 24 times the odds of a urine culture compared to a similar patient presenting to a physician with a lower propensity. This has important implications for resource use and patient care. Medical practice variations have been documented across a range of procedures and conditions [15], and have been explained in terms of clinical uncertainty and varying individual clinical thresholds at which physicians act [36].

A previous study in the United States reported that 67% of visits were associated with a prescription [37]. The lower proportion of 59% in our study could be due to prescriptions being provided but not filled in some circumstances, a general move towards more tolerance with a watch and wait approach, some prescriptions being filled outside our designated time window of 4 days, or inaccurate use of the cystitis code (for example, if it were used for asymptomatic bacteriuria). Despite *E*. *coli* resistance rates to ciprofloxacin over 20% during the study period [38], this was still the most frequently prescribed antibiotic (in 22% of cases). During this period, resistance to TMP-SMX was also above 20%, while resistance to nitrofurantoin was below 5% [38].

The implications of these findings on our understanding of community-level resistance rates should also be considered. Guidelines do not recommended urine cultures for uncomplicated cases; hence, we would expect to see an overrepresentation of complicated cases. However, we did not find that complicated cystitis were to be overrepresented–suggesting that resistance rates based on these cultures are not systematically over-estimating the level of antibiotic resistance. Nonetheless, the wide variation in practice among physicians complicates our understanding of resistance rates: we cannot reliably describe the patient population from whom urine cultures–and hence resistance rates–derive. Our understanding of resistance rates in the community should take this limitation of passive surveillance into account.

### Policy implications

The scope of variations in urine culturing described here suggests two potential implications: 1) urine cultures are not being used routinely when they apparently should be, and 2) physicians have practice styles that tend towards, or away from, culturing for cystitis, over and above basic clinical considerations. Antibiotic stewardship programs aim to support judicious and effective antibiotic use; an important component of this effort is the accurate diagnosis of infection and targeting of treatment. Optimization of resource use is always a concern, but is of particular significance in this era of antibiotic resistance: recommendations to expand the indications for additional urine culturing may be required as the epidemiology of UTIs shifts towards increasingly resistant pathogens. A greater understanding of the factors underlying the observed variations is required in order to promote adherence to (and adaptations as necessary of) recommendations.

A simple audit and feedback intervention targeting the top 20% of prescribers with feedback from England’s chief medical health officer showed promising results in reducing antibiotic prescriptions [35]. A similar intervention could be designed to address the use of cultures for urinary tract infections, adapted to include targeting of both potential over- and under-use of cultures. In British Columbia, family physicians are becoming more active in quality improvement activities. Understanding and reducing variations is one of the key topics of the professional development program for quality improvement, which is offered by the BC Patient Safety & Quality Council [39]. Efforts to address unwarranted variation would therefore appear to have traction within the profession, which will strengthen the possibilities for implementation and action.

A recent study demonstrated that laboratory-based feedback on indications for culturing changed culturing practices of physicians caring for nursing home [V. Leung, personal communication]. Selective reporting of microbiology results has been shown to influence antibiotic treatment selection [40]. Combining these ideas, laboratory reports to physicians could be designed to include general feedback about the indication for the specific culture (based on age and sex of the patient, for instance–which might reasonably be available in laboratory data), as well as a reminder of the appropriate indications for cultures in the general population.

Choosing Wisely initiatives aim to improve the quality of healthcare, in particular by reducing overuse of low-value services. Efforts to reduce overuse and underuse of urine cultures could integrate with the evaluation of these initiatives; for example, by following a framework to measure provider attitudes and awareness about the issue, assess unintended consequences of the campaign, and assess patient perceptions and outcomes [41].

In addition, sentinel physician networks–where a cohort of primary care physicians agrees to culture every patient with UTI symptoms seen in their practice–may be useful for surveillance of accurate antibiotic resistance rates. Alternatively, regular or periodic active surveillance studies may be required.

Finally, the results of this study suggest that there could be a benefit in re-stating clear and concise guidelines, even if the recommendations remain relatively unchanged.

### Limitations

This was an exploratory study, and, to the best of our knowledge, is the first time that privately held outpatient microbiology data on urine cultures has been linked to population-based physician service data. Undercounting (i.e., the failure to detect urine cultures associated with some cystitis visits) is an important consideration in our analysis, as LifeLabs is the largest but not the only outpatient laboratory service in the province. The market share is estimated to be approximately 60–62% province-wide, based on publicly available financial statements, during the time frame of this study [21]. Undercounting is a form of misclassification bias, and non-differential misclassification bias in the outcome generally results in bias toward the null [42]. It is unlikely that the presence of LifeLabs clinics, or indeed the compliance of a patient to proceed with sample submission, would differ systematically by the factors included in this analysis, other than by geographic region. As such, HSDA was included as a covariate in the models in an attempt to control for some of the coverage variability; however, the effect estimates for HSDA are themselves potentially biased and should not be directly interpreted (and as such, are not reported in the table). The sensitivity analyses demonstrated some minor changes in parameter estimates, but nothing that would alter the overall conclusions.

Patient variability was not modeled. Around half of individuals had more than one UTI visit during the study period. However, the complex structure of the data precluded the inclusion of both a physician and a patient random effect (i.e. the models did not converge). Patient visits are not nested within physicians. Therefore, any attempt to handle this would mean a manipulation of the data (e.g. forcing the nesting of the patients within physicians) and was not computationally feasible for our large study population.

The reason for the decrease in urine cultures in 2007 is unclear; no relevant change in policy or guidelines could be identified.

## Conclusion

This analysis suggests that physicians have highly variable tendencies to culture for cystitis, and the appropriate and efficient use of urine cultures can likely be improved. Further research is necessary to confirm these exploratory findings. However, effort directed towards both promoting the use of urine cultures in relevant cases, and restricting their use when not required, would not be misplaced. This effort could involve targeted audit and feedback to primary care providers and alignment with Choosing Wisely initiatives.

## Supporting information

S1 File A list of the data files and variables requested in the data extract. (DOCX)
