# Supplementary material for: What can we learn by examining variations in the use of urine culture in the management of acute cystitis? A retrospective cohort study with linked administrative data in British Columbia, Canada, 2005-2011
Source: PLoS One. 2019 Mar 8;14(3):e0213534. doi: 10.1371/journal.pone.0213534 (PMC6407775; doi:10.1371/journal.pone.0213534)
Supplement: S1 File — (DOCX) [file pone.0213534.s001.docx]

Data requested for the study *What can we learn by examining variations in the use of urine culture in the management of acute cystitis? A retrospective cohort study with linked administrative data in British Columbia, Canada, 2005-2011*

| **Data file** | **Time period** | **Variables** |
| --- | --- | --- |
| Medical Services Plan Payment Information (MSP) File | 01/04/2002 - 31/03/2012 | service date, service code, ICD9 diagnostic code, practitioner number (project specific), claim specialty code, client province, service where code, location of the service |
| Home and Community Care | 01/04/2002 - 31/03/2012 | client number (project-specified), absolute start date, absolute end date, provider category code, service code, service type code, provider ID (project-specific), provider health unit, provider is profit code, |
| Discharge Abstracts Database | 01/04/2002 - 31/03/2012 | BC hospital number (replaced by project-specific identification number), level of care, admission date, discharge date, admit category, entry code, discharge disposition, total length of stay, diagnosis (ICD-9 codes), diagnosis (ICD-10-CA codes), diagnosis type |
| PharmaCare | 01/04/2002 - 31/03/2012 | all plan types, date of service, practitioner number |
| Consolidation File | 01/04/2002 - 31/03/2012 | year of birth, month of birth, sex, neighbourhood income quintile/decile, health authority, health service delivery area, local health area, start day registered in year, total days registered in year |
| MSP Practitioner File | 01/04/2002 - 31/03/2012 | sex, year of birth, graduation location, year of graduation, membership status code, status start/change date, status end date, practitioner practice location |
| PharmNet MedHist | 01/04/2002 - 31/03/2012 | PHN (project-specific identification number), patient health authority, pharmacy identification number (project specific), pharmacy local health area, practitioner identification number (project specific), practitioner health authority, practitioner local health area, practitioner type, practitioner specialty flag, practitioner specialty type, DINPIN, Canadian brand name, chemical/generic drug name, drug strength, drug form units, dosage form code and description, date of service, quantity dispensed, days supply |
| LifeLabs | 01/04/2002 - 31/03/2012 | Patient identifier (project specific), date of culture, physician identifier (project specific), organism, susceptibilities |
| ACG case Mix Group (Johns Hopkins University) | 01/04/2002 - 31/03/2012 | ACG variables |
